# Structural Characteristics, Gelling Properties, *In Vitro* Antioxidant Activity and Immunomodulatory Effects of Rhamnogalacturonan-I Rich Pectic Polysaccharides Alkaline-Extracted from Wax Apple (*Syzygium samarangense*)

**DOI:** 10.3390/foods14071227

**Published:** 2025-03-31

**Authors:** Yue Lu, Siyu He, Zifan Zhao, Changxin Liu, Ye Lei, Mingyu Liu, Qing Zhang, Derong Lin, Yaowen Liu, Shang Lin, Xuesong Lu, Wen Qin

**Affiliations:** 1 College of Food Science, Sichuan Agricultural University, Ya’an 625014, China; luyue0212212@163.com (Y.L.); hesiyu_says@163.com (S.H.); 2024218014@stu.sicau.edu.cn (Z.Z.); z2037403620@163.com (C.L.); 19338641713@163.com (Y.L.); l15528553032@163.com (M.L.); zhangqing@sicau.edu.cn (Q.Z.); lindr2018@sicau.edu.cn (D.L.); lyw@my.swjtu.edu.cn (Y.L.); shanli@sicau.edu.cn (S.L.); 2 College of Culinary and Food Science Engineering, Sichuan Tourism University, Chengdu 610100, China

**Keywords:** *Syzygium samarangense*, pectic polysaccharides, gelling properties, antioxidant activity, immunomodulatory effects

## Abstract

To upgrade the utilization of *Syzygium samarangense* in food industries, the key biological component, i.e., polysaccharide, was extracted from the fruit by alkaline treatment, and its structural characteristics, physicochemical properties, gelling properties and biological activities were investigated. The findings demonstrated that the alkaline-extracted *S. samarangense* polysaccharide (SSP-AK) predominantly exists as a pectic polysaccharide with a high rhamnogalacturonan-I domain. The monosaccharide composition primarily includes rhamnose, glucuronic acid, galacturonic acid, glucose, galactose, and arabinose. The molecular weight distribution of SSP-AK was characterized by two peaks, with fraction 1 exhibiting a high molecular weight of 7658 kDa and fraction 2 exhibiting a molecular weight of 345.3 kDa. Meanwhile, SSP-AK exhibited excellent rheological behavior and gelling properties upon Ca^2+^-induced gelation, which may be related to its relatively low degree of esterification of 41.3%. Further studies revealed that higher concentrations of pectin and Ca^2+^ led to the formation of stronger gels. The SSP-AK gels exhibited superior rheological properties, increased hardness, enhanced water-holding capacity, and a more compact network structure than the other gels. Moreover, SSP-AK exhibited significant *in vitro* antioxidant activity and immunomodulatory effects, including significantly enhancing the DPPH and ABTS radical-scavenging abilities and production of NO, IL-6, and TNF-α in RAW264.7 cell models. This study enhances the understanding of *S. samarangense* cell wall polysaccharides and may facilitate their application in the development of functional and health-oriented food products.

## 1. Introduction

Wax apple (*Syzygium samarangense* Merr. and Perry) is a tropical evergreen tree predominantly cultivated in Southeast Asia, including Malaysia, Indonesia, Thailand, and China [1]. The fruit of *Syzygium samarangense* can be consumed fresh or processed into value-added products such as fruit juices, jams, wine, and phytomedicines, due to its rich nutritional components and therapeutic properties. A recent literature review summarized the various nutritional functions of *Syzygium samarangense*, including its pharmacological properties and antioxidant, anticancer, anti-inflammatory, antidiabetic, and hyperglycemic effects [1]. One of the primary biological components of *Syzygium samarangense* fruit is a natural polysaccharide, predominantly in the form of pectin. The structure of pectin generally comprises a smooth region, namely homogalacturonan (HG), and a hair region, namely rhamnogalacturonan-I (RG-I) and rhamnogalacturonan II (RG-II) [2]. HG consists of a linear chain of α-1,4-linked D-galacturonic acid (GalA), with some GalA residues being methyl esterified or acetylated [3]. RG-I has a backbone of repeating disaccharide units of α-1,4-linked GalA and α-1,2-linked L-rhamnose (Rha) [3,4]. The side chains of RG-I are often composed of galactose (Gal) and arabinose (Ara), resulting in a branched structure [3,4]. To the best of our knowledge, the understanding of *Syzygium samarangense* pectic polysaccharides, including their structural characteristics and biological activities, such as antioxidant activity and immunomodulatory effects, remains limited, thereby affecting their potential application as healthy food ingredients.

One of the most important features of pectin is its gelling properties, and pectin hydrogels are utilized as food thickeners and good gels. Pectin can be divided into two types based on its DE, i.e., high-methyl pectin (HMP, DE > 50%) and low-methyl pectin (LMP, DE < 50%). These two types of pectin are believed to result in two kinds of pectin gelation mechanisms. For HMP, optimal gelation occurs at a low pH (2.5–3.5) and high sugar concentrations (55–75%) [5]. Low pH reduces the electrostatic repulsion between pectin molecules, while sugar decreases water availability, promoting intermolecular cross-linking and forming a stable gel. In contrast, LMP relies on calcium ions for cross-linking, with calcium binding to carboxyl groups in pectin molecules to form an “egg-box” structure that stabilizes the gel network [6]. Due to the absence of added sugar, calcium-induced pectin gelation is receiving increasing attention in the health food industry. Both calcium and pectin concentrations are believed to affect pectin gelation. For instance, a low calcium concentration results in a weak gel, while excessive calcium can cause the gel to become overly rigid [7]. Hence, to form a satisfactory pectin gel, a proper calcium concentration and pectin concentration should be considered. Most research on pectin gelation has focused on the HG domain; however, recent studies have reported that the RG-I domain also influences pectin gelation. The side chains of the RG-I domain play a vital role in pectin gelation. For instance, the removal of arabinan and galactan side chains can lower the gelation capacity of pectin [8,9,10]. Another advantage of utilizing RG-I-rich pectin is its greater biological activity than that of the HG domain. Despite the reported bioactivities of *Syzygium samarangense*, further studies on the nutritional functions of its pectic polysaccharides are required. 

Due to the limited understanding of *Syzygium samarangense* fruit pectin, its gelation capacities and biological activities remain unknown. Therefore, the extraction yield, structural characteristics, and gelling properties of alkaline-extracted *Syzygium samarangense* pectic polysaccharides were investigated. Additionally, the effects of pectin concentration and Ca^2+^ concentration on the gelation of *Syzygium samarangense* pectic polysaccharides were investigated. Finally, we also demonstrated the *in vitro* antioxidant activity and immunomodulatory effects of the extract, suggesting its potential applications in both food gels and healthy food ingredients.

## 2. Materials and Methods

### 2.1. Samples and Chemicals

*Syzygium samarangense* fruits were obtained from Fuzhou Jingpin Jinbao Agricultural Technology Co. Ltd. (Fuzhou, China). The samples were washed, freeze-dried, and stored at −20 °C for further use. All chemicals were of analytical grade unless otherwise specified and purchased from Sigma-Aldrich (Burlington, MA, USA).

### 2.2. Alkaline Extraction of Syzygium samarangense Pectic Polysaccharide

The method for the alkali extraction of wax apple polysaccharides was slightly modified based on Cui et al. [11]. The *Syzygium samarangense* fruit powder was soaked in 95% ethanol for 4 h to remove polyphenols and reducing sugars. The supernatant was discarded after centrifugation at 7000 rpm for 20 min, and the pellet was extracted with 6 M NaOH (pH was adjusted to 10) at a ratio of 1:30 (*w*/*v*) at 80 °C for 2 h. The supernatant was neutralized with 1 M HCl. The slurry was centrifuged, and the supernatant was de-starched by the addition of heat-stable α-amylase (Beijing Solarbio Science & Technology Co. Ltd., Beijing, China) at 90 °C for 4 h until the KI-I assay was negative. Subsequently, the protein in the supernatant was removed using pancreatin protease at 40 °C for 10 h. The enzymes were inactivated by heating at 90 °C for 120 min, and the mixture was then centrifuged. The supernatant was pooled, concentrated, and precipitated overnight with three volumes of 95% ethanol at 4 °C. The precipitate was dissolved in Milli-Q water and then dialyzed for 2 days using a 3.5 kDa cut-off dialysis bag. Afterwards, the samples were freeze-dried and stored at −20 °C for further analysis and experiments, and this sample was referred to as SSP-AK. 

It should be noted that *Syzygium samarangense* polysaccharides were also extracted by other methods for comparison with alkaline-extracted polysaccharides. Each extraction methods were described as follows,

Hot water extraction: The pellet was extracted with Milli-Q water at a ratio of 1:30 (*w*/*v*) with continuous stirring at 90 °C for 4 h twice. This sample was referred to as SSP-H. 

Autoclave extraction: The pellet was extracted with Milli-Q water at a ratio of 1:50 (*w*/*v*) using an autoclave (GI54DS, Zeal Way, Xiamen, China) at 121 °C and 0.12 MPa for 20 min. This sample was referred to as SSP-AU.

Ultrasound-assisted extraction: The pellet was extracted with Milli-Q water at a ratio of 1:30 (*w*/*v*) for 20 min twice at a power of 400 W using an ultrasonic homogenizer (JY92-IIN, Ningbo Scientz Biotechnology Co. Ltd., Ningbo, China). This sample was referred to as SSP-U.

Microwave-assisted extraction: The pellet was extracted with Milli-Q water at a ratio of 1:30 (*w*/*v*) for 10 min twice at a power of 420 W using a laboratory microwave oven (MKJ-J1-3, Qingdao microwave applied technology Co. Ltd., Qingdao, China). This sample was referred to as SSP-M.

Acidic extraction: The pellet was extracted with 1 M citric acid (pH adjusted to 2.5) at a ratio of 1:30 (*w*/*v*) at 80 °C for 2 h. The supernatant was neutralized using 1 M NaOH. This sample was referred to as SSP-AC. 

Deep eutectic solvent (DES) extraction: The DES was prepared by mixing choline chloride and urea at a ratio of 1:3 with a water content of 30%. The pellet was extracted with DES at a ratio of 1:30 (*w*/*v*) at 80 °C for 2 h. This sample was referred to as SSP-DES.

### 2.3. Determination of Total Polysaccharide and Total Protein Content of SSPs

The total polysaccharide content was determined, and the total protein content of the SSP sample was determined according to a previous study [12].

### 2.4. Carbohydrate Analysis

Monosaccharide composition: The monosaccharide composition of SSPs was analyzed using high-performance liquid chromatography (HPLC) equipped with a diode array detector (DAD) [12]. In brief, 5 mg/mL of each SSP sample solution was hydrolyzed with 4 M trifluoroacetic acid (TFA) for 10 h. After the removal of TFA, the hydrolysates were dissolved in 1 mL of Milli-Q water and derivatized with a 0.5 M phenyl-3-methyl-5-pyrazolone (PMP) methanol solution. Each sample (20 µL) was injected onto the column (InfinityLab Poroshell 120 EC-C18, 4 µm, 4.6 × 150 mm, Agilent Technologies, Inc., Palo Alto, CA, USA) with an LC system (Agilent 1260, Agilent Technologies, Palo Alto, CA, USA) and analyzed using the following DAD system at a wavelength of 245 nm. The elution buffer consisted of 83% 0.1 M PBS (pH 6.7) and 17% acetonitrile, the flow rate was 1.0 mL/min, and the column temperature was 30 °C. Monosaccharide standards, including rhamnose, mannose, galacturonic acid, glucuronic acid, galactose, glucose, xylose, and arabinose, were analyzed as described above.

The HG domain content was calculated as HG = GalA − Rha [13]. The RG-I domain content was calculated using the formula RG-I = 2 Rha + Ara + Gal [13].

Molecular weight: The molecular weight distribution (M_w_) and polydispersity (M_w_/M_n_) of the SSPs were determined using high-performance size exclusion chromatography coupled with multi-angle laser light scattering and a refractive index detector (HPSEC-MALLS-RID, Wyatt Technology Co., Santa Barbara, CA, USA) according to a previous study [14]. Briefly, 100 µL of the SSP sample solution (2.5 mg/mL in elution buffer) was injected onto the column (OHpak SB-806M HQ, 8.0 × 300 mm, Resonac Chemistry, Tokyo, Japan) with an Agilent 1260 LC system and analyzed using a laser detector (Wyatt Technology, Santa Barbara, CA, USA) and an Optilab rEX refractometer (Wyatt Technology, Santa Barbara, CA, USA). The elution buffer was a 0.9% NaCl solution with 0.02% NaN_3_ at a flow rate of 0.5 mL/min, and the column temperature was set to 35 °C.

Fourier transform infrared (FT-IR) spectroscopy: FT-IR analysis was carried out with a recording in the frequency range of 4000–500 cm^−1^ using a Spectrum 100 FT-IR (PerkinElmer, Waltham, MA, USA) instrument [12].

Degree of esterification (DE): The degree of esterification (DE) was determined according to a previous report with minor modifications [15]. In brief, 1–2 drops of 1% phenolphthalein were added to a 5 mg/mL SSP sample solution and then titrated with 0.1 M NaOH to give a reddish color to determine the non-esterified carboxylic groups (*v*_1_). Subsequently, 0.5 mL of 0.5 M NaOH was added, followed by continuous stirring for 30 min for the saponification of esterified groups of the SSP sample. The mixture was neutralized with 0.5 mL of 0.25 M HCl until the red color turned clear. Then, 1–2 drops of phenolphthalein were added again, and titration with 0.1 M NaOH was carried out. The volume was recorded to represent the esterified carboxylic groups (*v*_2_). DE was calculated using the following equation:

$$\mathrm{DE}\left(\%\right)=\frac{v_{2}}{v_{1}+v_{2}}\times 100\%$$

### 2.5. Gelling Properties

#### 2.5.1. Preparation of Ca^2+^-Induced Gel

The Ca^2+^-induced gelation of SSP sample was carried out according to the literature with some modifications [7]. Each SSP sample obtained using the different extraction methods was dissolved in Milli-Q water at a concentration of 10 mg/mL, and the pH was adjusted to 3.0 using 0.5 M HCl. Gelation was performed by adding 0.99 mg/mL CaCl_2_ solution to the SSP solution in a total volume of 5 mL with continuous shaking at 25 °C for 30 min. The prepared gels were immediately used for rheological behavior analysis. The appearance of each gel sample was recorded using photography.

#### 2.5.2. Effect of Pectin Concentration and Ca^2+^ Concentration on Gelling Properties of SSP-AK Sample

The SSP-AK sample was dissolved in a series of concentrations i.e., from 0.55 to 1.32%, and was added with a series of concentrations of CaCl_2_ solutions at a pH of 3.0. Gelation was carried out at 25 °C for 30 min with continuous shaking, and the prepared gels were immediately used for further analysis.

#### 2.5.3. Rheological Behaviors

The rheological behaviors of each SSP gel sample were analyzed using a modular compact rheometer (MCR 102e, Anton Paar GmbH, Graz, Austria) equipped with a parallel plate at 25 °C (50 mm diameter). The strain sweep measurement was conducted across a strain range of 0.01–100% with a fixed frequency of 1 Hz after equilibrating at 25 °C for 2 min. The relationship between the storage modulus (G′), loss modulus (G″), and strain was recorded. A frequency sweep was conducted across a frequency range of 0.1–10 Hz with a fixed strain of 0.1%, and the relationship between G′, G″, and frequency was determined. The time sweep was conducted across a time range of 0–60 min with a fixed frequency of 1 Hz and strain of 0.1%, and the relationship between G′ and G″ and time was recorded. The relationship between viscosity and shear rate was measured after equilibrating at 25 °C for 2 min and applying shear rates of 1 to 100 s^−1^.

#### 2.5.4. Water-Holding Capacity

The water-holding capacity (WHC) of each SSP gel sample was determined using a previously reported method with minor modifications [16]. In brief, 2.0 g of gel was centrifuged at 10,000 rpm for 10 min at room temperature, and the supernatant was removed. The total weight of centrifuge tube and gel sample was recorded before and after centrifugation, and the WHC was calculated as the equation as follows,

$$\mathrm{WHC}\left(\%\right)=\frac{W_{2}-W_{0}}{W_{1}-W_{0}}\times 100\%$$

where W_0_ is the weight of the centrifuge tube without the gel sample, and W_1_ and W_2_ are the weights of the centrifuge tube with the gel sample before centrifugation, and after centrifugation.

#### 2.5.5. Gel Hardness

The gel hardness of each SSP gel sample was measured using a texture analyzer (TA.XT.Plus, Stable Micro System, Surrey, UK) equipped with a cylinder probe p/0.5 [7].

#### 2.5.6. Scanning Electron Microscopy (SEM) Analysis

The SEM analysis was carried out by using a Merlin tabletop microscope (ZEISS, Oberkochen, Germany) [7]. The freeze-dried gel samples were coated with a gold-palladium layer, and the microstructure was observed under an accelerating voltage of 3.0 kV.

### 2.6. Antioxidant Activity of SSP-AK

The DPPH and ABTS radical-scavenging activities of SSP-AK were determined according to a previously reported method [17,18]. The DPPH radical-scavenging ability of SSP-AK was determined by incubating 1 mL of DPPH solution (0.35 mM in 50% ethanol) with 125 µL of SSP-AK solution at different concentrations. After incubation at 37 °C in the dark for 30 min, the absorbance of each sample at 517 nm was measured, and the DPPH radical-scavenging capacity was calculated. The ABTS radical-scavenging ability was determined by incubating o1 mL of ABTS solution with 200 µL of SSP-AK at different concentrations. After the reaction at 37 °C in the dark for 10 min, the absorbance at 734 nm was measured, and the ABTS radical-scavenging ability was calculated. 

### 2.7. Immunomodulatory Activity of SSP-AK

The *in vitro* immunomodulatory activity of SSP-AK was determined using a RAW 264.7 macrophage model, as previously described [14]. RAW 264.7 cells were cultured in 5% CO_2_ at 37 °C, and the cell viability of SSP-AK samples at different concentrations was assayed using the MTT colorimetric method, as described previously [12]. The production of nitric oxide (NO) and cytokines, such as tumor necrosis factor-alpha (TNF-α) and interleukin-6 (IL-6), was measured using an ELISA kit (eBioscience, San Diego, CA, USA) [12].

### 2.8. Statistics Analysis

All assays were carried out in triplicate. One-way analysis of variance (ANOVA) was performed on the obtained data using the Statistical Products and Services Solutions (SPSS) software (26.0, IBM, Albany, NY, USA). Duncan’s multiple range test was used to analyze the variance of the experimental group. When the *p*-value was <0.05, the average was considered different.

## 3. Results and Discussions

### 3.1. Chemical Components and Monosaccharide Composition of SSPs

The chemical components and extraction yield of polysaccharides are generally affected by the extraction method. Hence, we compared the effects of different extraction methods, including hot water extraction, citric acid extraction, DES extraction, autoclave extraction, ultrasound-assisted extraction, and microwave-assisted extraction, on *Syzygium samarangense* polysaccharides. SSP-AK had the highest extraction yield of 5.3% compared to other extraction methods, which was approximately 3.5-fold higher than the SSP sample extracted by hot water extraction (Table 1). Meanwhile, SSP-AK also showed the highest total polysaccharide content of 88.4% (Table 1). Taken together, these results suggest that alkaline extraction is the most efficient method for extracting *Syzygium samarangense* polysaccharides. 

As shown in Table 1, it was observed that SSP-AK was identified to contain six monosaccharides: rhamnose (Rha), glucuronic acid (GluA), galacturonic acid (GalA), glucose (Glu), galactose (Gal), and arabinose (Ara). GalA, Gal, and Ara were the predominant monosaccharides, suggesting SSP-AK was a typical pectic polysaccharide. GluA was a minor component among the monosaccharides, suggesting that it plays a limited role in the overall physicochemical properties and biological activities of the polysaccharide (Table 1). To the best of our knowledge, the study by Wang et al. is the only existing research on the monosaccharide composition of *Syzygium samarangense* fruit polysaccharides [19]. The study concluded that rhamnose (Rha), arabinose (Ara), xylose (Xyl), mannose (Man), glucose (Glu), and galactose (Gal) were present, with Xyl and Glu being the most abundant monosaccharides. Our findings differ significantly from those of previous studies, particularly regarding the composition and relative abundance of monosaccharides. We believe that these discrepancies may be explained by the fact that we extracted different types of polysaccharides from *Syzygium samarangense* fruit. The SSP samples extracted using other extraction methods showed a similar monosaccharide composition but differed in the molar ratio of each monosaccharide (Table 1). SSP-AK showed a predominantly high proportion of RG-I domain of 64.8%, while the proportion of HG was around 22.6%, indicating that SSP-AK is an RG-I rich pectic polysaccharide. The molar ratio between (Ala + Gal) and Rha reflects the side chains, that is, the branching size in the RG-I domain, which is critical for determining the structural complexity of pectic polysaccharides. The branching size of SSP-AK was 7.6, which was lower than that of the SSP sample obtained by hot water extraction (Table 1). The decreased branched sizes observed in SSP-AK suggest that alkaline extraction led to the degradation of neutral side chains in the RG-I domain during extraction. Nevertheless, the monosaccharide composition results suggested that the RG-I domain of SSP-AK was highly branched by arabinan, galactan, or arabinogalactan side chains.

The HG domain of pectin can be methyl esterified, and we found that the degree of esterification (DE) of SSP-AK was 41.3% (Table 1), which can be considered as LMP (DE < 50%). The DE of SSP-AK was significantly lower than that of the SSP sample obtained using other extraction methods, particularly hot water extraction (Table 1). This result is consistent with the report that alkaline treatment may contribute to the de-esterification of pectin [8,20,21].

### 3.2. Molecular Weight Distribution of SSPs

**Figure 1:**
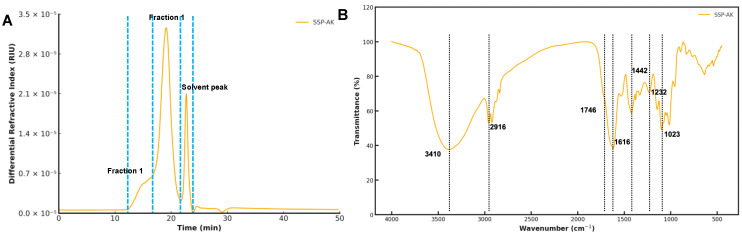
(**A**) Size exclusion chromatogram and (**B**) FT-IR spectra of SSP-AK.

As shown in Table 2 and Figure 1A, SSP-AK exhibited a molecular weight distribution with two peaks. Fraction 1 of SSP-AK had a high M_w_ of 7658 kDa with a mass fraction of 15.7%, while the major fraction, Fraction 2 of SSP-AK, had a Mw of 345.3 kDa (Table 2). We observed that the molecular weight distribution of the SSP sample was significantly affected by the extraction method (Figure S1A–G). The major fraction of SSP-AK was half of the SSP sample obtained by hot water extraction of 741.5 kDa, suggesting that alkaline extraction may break down the polysaccharide (Table 2 and Figure S1A).

### 3.3. FT-IR Analysis of SSP-AK

The FT-IR spectra shown in Figure 1B display the characteristic absorption peaks of SSP-AK. It should be noted that the FT-IR results did not vary in SSP samples obtained by different extraction methods (Figure S1H). The board bands around 3410 and 2916 cm^−1^ were the typical bands of hydroxyl groups in polysaccharides. The peaks at 1746 cm^−1^ were attributed to esterified GalA due to the stretching of the carboxylic groups of esterified GalA [18]. The difference in the peaks at 1746 cm^−1^ for distinct SSPs samples indicates their DE abundance. The intense peak at around 1616 cm^−1^ was attributed to the C=O asymmetric stretching of –COO, suggesting the presence of uronic acids [12,22]. Additionally, the absorption band near 1442 cm^−1^ was attributed to the presence of carboxyl groups [23].

### 3.4. Rheological Properties and Gelling Properties of SSP-AK

**Steady-state flow behavior**

Rheological properties and gelling properties are key features of pectic polysaccharides. Our results demonstrated that the apparent viscosity of SSP-AK solutions decreased as the shear rate increased from 0.1 to 100 s^−1^ (Figure S2A). The results indicated that the SSP-AK solution had a shear-thinning behavior in aqueous solution at a concentration of 1% and was a non-Newtonian pseudoplastic fluid [24]. Meanwhile, SSP samples obtained using other extraction methods showed similar flow behavior in the same shear rate range (Figure S2A). SSP-AK exhibited the highest apparent viscosity within the shear rate range of 0.1–2.24 s^−1^ compared to other SSP samples, which then rapidly decreased to below 0.013 Pa·s as the shear rate increased (Figure S2A). This finding indicates that SSP-AK could retain a relatively high viscosity under low shear stress, but its viscosity did not vary significantly from those of other SSP samples under high shear stress. The storage modulus (G′) and loss modulus (G″) of SSP-AK during the time-sweep test are shown in Figure S2B. SSP-AK had higher G′ values than G″ values during the 60 min time-sweep test, suggesting that it was in a more colloidal state [25]. The stable and high G′ value of SSP-AK suggested that this polysaccharide solution was in a stable colloidal state, which is consistent with the higher apparent viscosity described above (Figure S2A). The differences in rheological properties i.e., apparent viscosity, G′ and G″ values of distinct SSP samples may be related to their differences in M_w_ and branching degree [26].

**Gelling properties**

The gelling properties of SSP-AK were further investigated. The apparent viscosity of the SSP-AK solution (1%) increased by approximately 100-fold after Ca^2+^-induced gelation (Figure S2D). Interestingly, SSP-AK appeared to be the only SSP sample that could form a stable gel, while the SSP samples extracted by other methods tended to form liquid-like fluids (Figure S2C). As expected, SSP-AK had the highest viscosity among all SSPs gels. At a shear rate of 1.12 s^−1^, the apparent viscosity was 16.1 Pa·s, which was 400~1100-fold higher than that of the other gels. Taken together, SSP-AK formed a stable gel, while the other SSP samples tended to form weak gels. The gelling properties are believed to be highly correlated with the degree of esterification. The DE of SSP-AK was the lowest at 41.3%, whereas all other SSP samples had DE greater than 50% (Table 1). LMP can form a gel following the egg-box model under the induction of Ca^2+^, whereas HMP, i.e., SSPs samples with more than 50% DE, tend to only form gels in the presence of acid and sugars [27,28]. The satisfactory gelling ability of SSP-AK may also be explained by the relatively high proportion of RG-I as 64.8% (Table 1). It has been reported that the neutral sugar sidechains of the RG-I region of pectin can improve the gel network and thereby strengthen the gel [29]. Taken together, these results indicate that SSP-AK is a suitable polysaccharide for pectin hydrogel development.

### 3.5. Effect of Pectin Concentrations on Gelling Properties of SSP-AK

**Steady-state flow behaviors**

**Figure 2:**
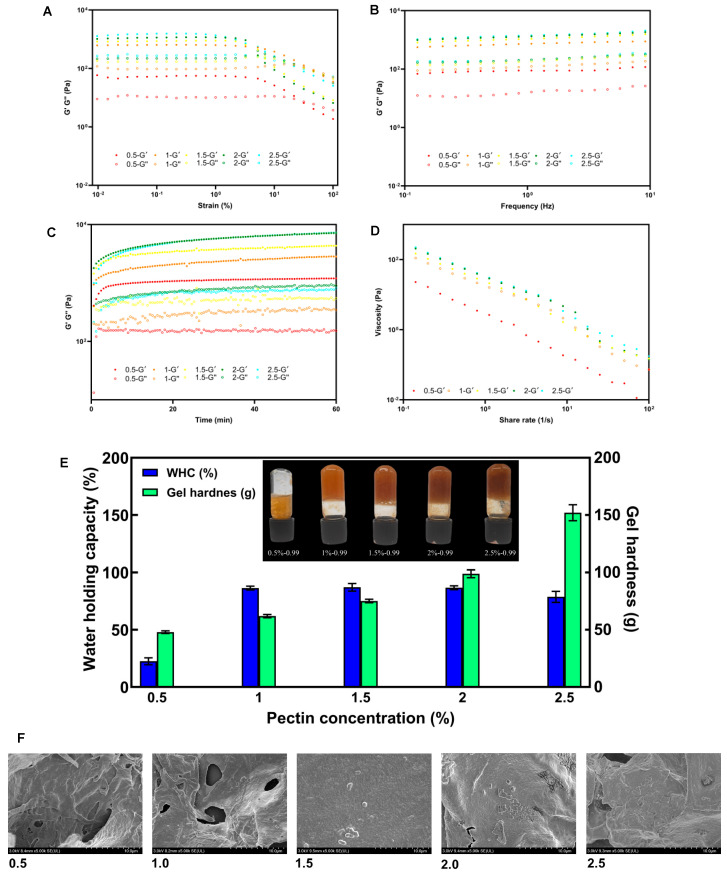
(**A**–**D**) Strain sweep, frequency sweep, time sweep, flow behaviors of SSP-AK prepared by different pectin concentrations (0.5–2.5%); (**E**) WHC, gel hardness and the images of SSP-AK gel samples by different pectin concentrations; (**F**) SEM of SSP-AK gel samples by different pectin concentrations.

To upgrade SSP-AK as a potential food gel and thickener, the effects of pectin concentration on its gelling properties were further investigated. The flow behavior results suggested that all SSP-AK gel samples behaved as non-Newtonian pseudoplastic fluids. During the stress sweep test shown in Figure 2A, in the low-strain region, the G′ value was always higher than the G″ value of all gel samples, indicating gel formation of SSP-AK solutions at concentrations ranging from 0.5% to 2.5%. As expected, the increased SSP-AK concentration resulted in a significantly higher G′ value. The linear viscoelastic region (LVR) reflects the stability of the gel samples under stress, where the G′ value remains stable. SSP-AK solutions with concentrations of 0.5% and 1.0% showed similar and widest LVR of around 0.01% to 1.47%, while the LVR of gels decreased to 0.46% as the SSP-AK concentration increased to a maximum of 2.5%. The G′ continuously decreased with increasing strain, and the yield point, where G′ exceeds G″, is considered to represent the stability of the gels. The gel behaves solid-like when the strain value is below the yield point, and the network is disrupted, causing it to behave more liquid-like when the strain is higher than the yield point [30]. The highest yield point was found in the SSP-AK gel with a concentration of 1.0%, at around 46%, followed by the SSP-AK gel with a concentration of 0.5%, at around 31%. The increase in pectin concentration resulted in a lower yield point; for instance, the yield point of SSP-AK with a concentration of 2.5% was 21%. In the frequency sweep test, the strain was set to 1% to ensure that the gel remained within the LVR. As shown in Figure 2B, all gel samples had a consistently greater G′ value than G″ value within the frequency range of 0.1–10 Hz, indicating the formation of a stable elastic gel. The G′ and G″ increased with higher pectin concentrations, which may be related to the increased cross-linking connection regions between pectin chains [8]. However, there were only minor differences in both G′ and G″ among SSP-AK gels with concentrations ranging from 1.5% to 2.5%, indicating that the saturated pectin concentration may have been reached. In the time-sweep test, all gel samples showed an increase in both G′ and G″ values in the first 10 min (Figure 2C). In addition, except for the gel with a concentration of 0.5%, the G′ and G″ values showed a slight upward trend with increasing time, which suggests a longer gelation time could strengthen the gel network and improve the gel stability of SSP-AK gels. As expected, G′ and G″ values were higher when the pectin concentration increased. However, no significant difference in G′ and G″ was observed between gels with a concentration of 2.0% and 2.5%. Figure 2D shows that the SSP-AK gel with a concentration of 0.5% had the lowest apparent viscosity at shear stress levels from 0.1–100 s^−1^, while the gel with 1.0% concentration had a slightly lower apparent viscosity compared to other gel samples. Taken together, the increase in pectin concentration in the SSP-AK gel could strengthen the gel network, improve gel stability, and increase viscosity. It should also be noted that an oversaturated pectin concentration may not enhance gel strength.

**Gel appearance and physicochemical properties**

Figure 2E shows images of SSP-AK gel samples with different pectin concentrations. The color of the gels gradually darkened with increasing pectin concentration, which may be explained by a more compact gel network connection. Meanwhile, the gel with a concentration of 0.5% did not form a strong gel and could not suspend in the vial, while other gels with higher concentrations, i.e., 1.0–2.5%, formed a stable solid-like gel and were more evenly distributed. This phenomenon is consistent with their rheological properties, such as apparent viscosity and G′ values, as described above. 

The physicochemical properties of the gel samples, including gel hardness and water-holding capacity (WHC) were further examined. As shown in Figure 2E, gel hardness increased with increasing pectin concentration of SSP-AK. At a concentration of 0.5%, the gel hardness was 47.9 g, which gradually increased to 152.1 g as the pectin concentration increased to 2.5%. It has been reported that the formation of hydrogen bonds and hydrophobic interactions between pectin molecules is insufficient to establish a robust network gel structure when the pectin concentration is low [31]. In contrast, an increased pectin concentration could result in a large number of available binding sites and inter-chain hydrogen bonds, which significantly enhanced gel strength [8,31,32]. Meanwhile, the 0.5% SSP-AK gel exhibited a poor WHC of 22.6%, indicating a less stable gel network. The gel with a 1.0% concentration had a 3.8-folded higher WHC of 86.4% and did not differ significantly among the gels with 1.5–2% concentrations. This result is consistentwith the previous literature, which suggested that further increase in pectin concentration did not significantly enhance the WHC of gels [8].

**Microstructure**

The SEM results showed the microstructures of SSP-AK gels with different pectin concentrations (Figure 2F). At low pectin concentrations (0.5 and 1 mg/mL), large pores were observed in SSP-AK gels. This may be explained by the less extensive cross-linking between pectin molecules and calcium, resulting in a looser network structure and larger pores. With an increase in pectin concentration, the SSP-AK gel exhibited a smoother surface with fewer and smaller pores. The network became more compact, indicating greater cross-linking, which is in accordance with the higher gel strength and stability described above.

### 3.6. Effect of Ca^2+^ Concentrations on Gelling Properties of SSP-AK

**Steady-state flow behaviors**

**Figure 3:**
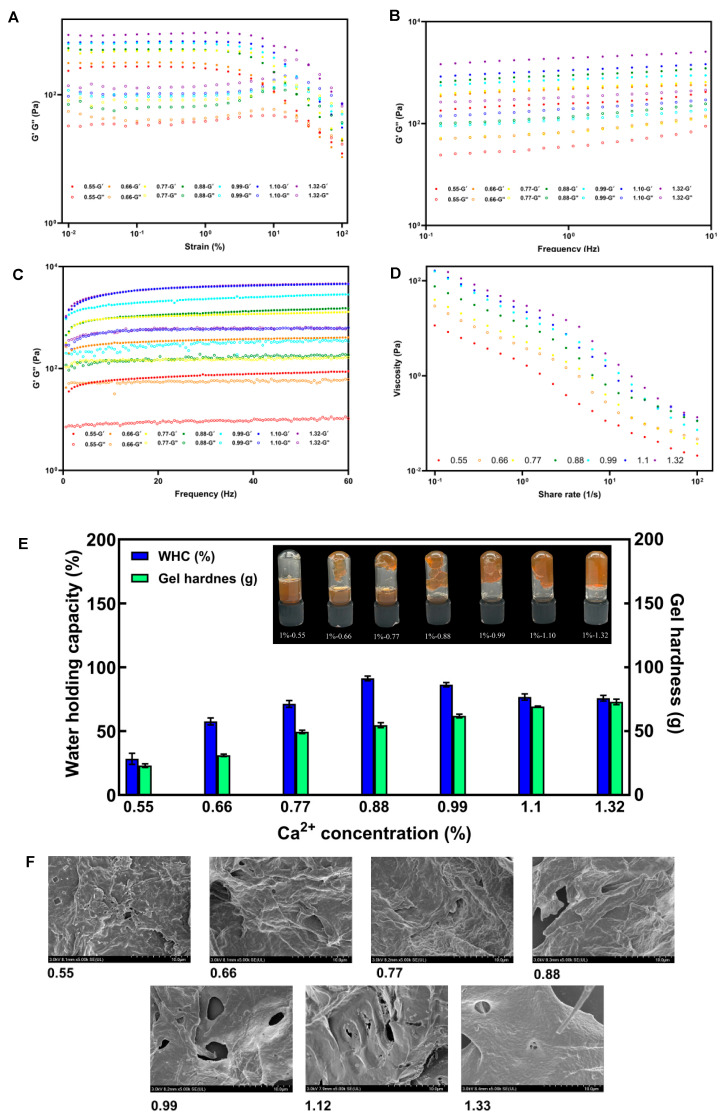
(**A**–**D**) Strain sweep, frequency sweep, time sweep, flow behaviors of SSP-AK prepared by different Ca^2+^ concentrations; (**E**) WHC, gel hardness, and the images of SSP-AK gel samples by different Ca^2+^ concentrations; (**F**) SEM of SSP-AK gel samples by different Ca^2+^ concentrations.

The gelling properties of SSP-AK gels induced by different Ca^2+^ concentrations were examined, and the results are shown in Figure 3. As expected, the addition of Ca^2+^ concentrations significantly affected the flow behavior of SSP-AK gels. In the strain sweep test, all gel samples had a greater G′ than G″ at low stress, indicating that all gels were in the solid phase. The gel with 0.55 mg/g Ca^2+^ showed the narrowest LVR, ranging from 0.01% to 1.47%, and the LVR became wider as the Ca^2+^ concentration increased from 0.55 to 1.32 mg/g. The widest LVR was found in gels with Ca^2+^ concentrations of 1.10 and 1.32 mg/g, ranging from 0.01% to 14.7%. Interestingly, the highest yield point was observed in the gel with 0.55 mg/g Ca^2+^, at around 47.1%, whereas the other gel samples showed lower yield points. For instance, the yield point of the gel with 0.66% and 0.88 mg/g Ca^2+^ was 21.6%. A similar trend, where an increase in Ca^2+^ concentration led to increased G′ values in gel samples, was observed in both frequency and sweep and time sweeps (Figure 3A–D). Furthermore, the gel with higher Ca^2+^ concentration also showed a greater apparent viscosity at the shear rate range of 0.1–100 s^−1^. Taken together, the addition of more Ca^2+^ could induce a stable gel with better flow behavior, which is consistent with previous studies showing that increasing Ca^2+^ concentration enhances the gel network [8,31,32].

**Gel appearance and physicochemical properties**

As shown in Figure 3E, gels with low Ca^2+^ concentrations (0.55–0.88 mg/g) were unable to form strong and stable gels. At a concentration of 0.55 mg/g, the gel failed to adhere to the vial walls, but as the Ca^2+^ concentration increased to 0.88 mg/g, a larger fraction of the gel could be suspended within the vial. It appears that only gels with Ca^2+^ concentrations above 0.99 mg/g could form a stable and strong gel structure. Additionally, the gel hardness increased from 23.1 to 72.9 g as the Ca^2+^ concentration increased (Figure 3E). It should be noted that the changes in gel hardness were more significant at low Ca^2+^ concentration ranges. The WHC of gels increased from 28.4% to 91.3% with the increase in Ca^2+^ concentration from 0.55 to 0.88 mg/g, and the WHC slightly decreased to 75.8%. This result is in accordance with the gel appearance, where only gels with high Ca^2+^ concentrations could form a stable gel structure.

**Microstructure**

The SEM results showed the microstructures of SSP-AK gels with different Ca^2+^ concentrations (Figure 3F). At low Ca^2+^ concentrations (0.55–0.88 mg/g), the gel structure of SSP-AK gels appeared loose, with a less dense network showing numerous pores and larger voids, indicating weak cross-linking. The gel structure became denser with increasing in Ca^2+^ concentration. However, large pores were still observed (Figure 3F). At a Ca^2+^ concentration of 1.32 mg/g, the gel had a smooth surface and compact structure, indicating a very stable gel.

### 3.7. Antioxidant Activity of SSP-AK

**Figure 4:**
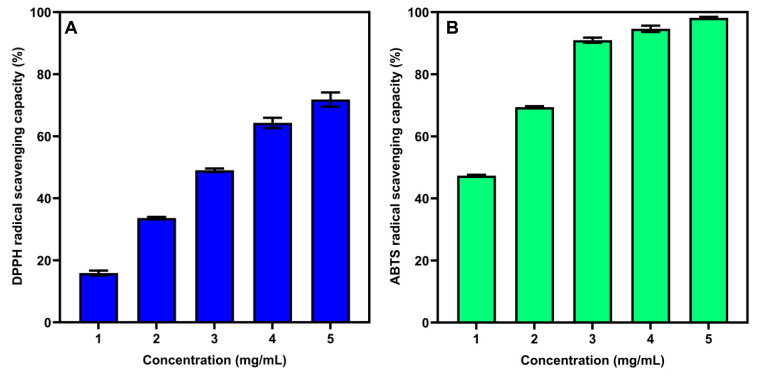
(**A**) DPPH radical-scavenging activity and (**B**) ABTS scavenging activity of SSP-AK.

Many studies have demonstrated that natural polysaccharides play an important role in antioxidant activity [33,34]. Hence, in the present study, we examined the *in vitro* antioxidant activity, i.e., the DPPH and ABTS radical-scavenging ability of SSP-AK. As shown in Figure 4, SSP-AK exhibited good DPPH and ABTS radical-scavenging abilities in a dose-dependent manner at a polysaccharide concentration of 1–5 mg/mL. The DPPH radical-scavenging activity ranged from 15.9% to 71.8%, with an IC_50_ value of 2.84 mg/mL, while the ABTS radical-scavenging activity ranged from 47.3% to 98.2%, with a lower IC_50_ value of 1.05 (Figure 4). Generally, the mechanism is estimated that the presence of electrophilic groups like keto or aldehyde, in acidic polysaccharides, facilitates the liberation of hydrogen from O-H bonds, and these groups can improve the radical-scavenging activities [33,35]. Our results indicated that SSP-AK exhibited satisfactory antioxidant activity and may be used as a natural antioxidant source.

### 3.8. Immunomodulatory Activity of SSP-AK

**Figure 5:**
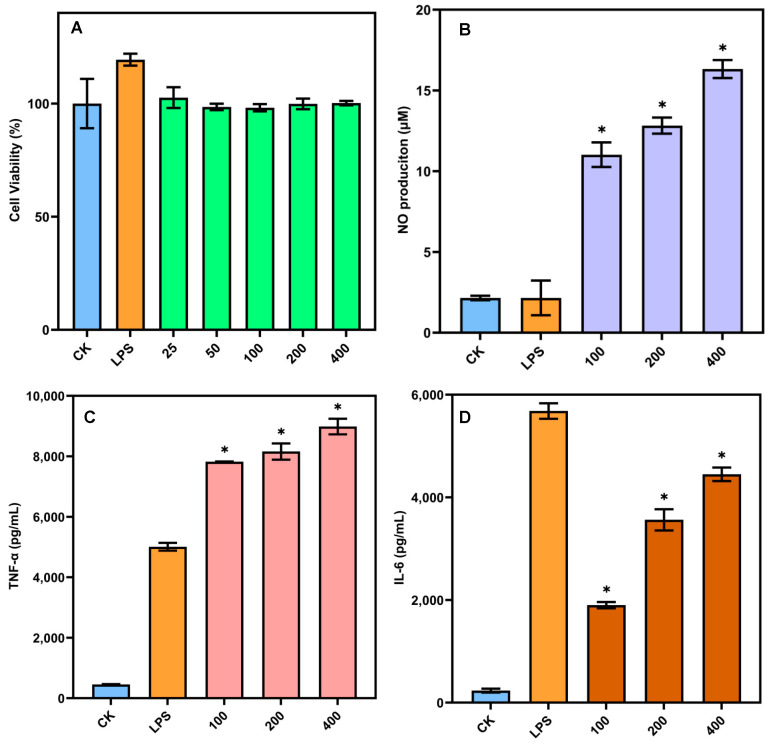
(**A**–**D**) Cell viability, NO production, TNF-α production and IL-6 production of SSP-AK. CK is the blank control group; LSP is the positive control group (lipopolysaccharide). * stands for a significant difference (*p* < 0.05).

One of the most vital health-promoting functions of natural polysaccharides is their immunomodulatory activity [36,37,38]. Thus, to examine the immunostimulatory activity of SSP-AK, macrophages were selected as the suitable model for *in vitro* studies. As shown in Figure 5A, the MMT assay indicated that LPS (1 µg/mL) and SSP-AK at concentrations ranging from 25–400 µg/mL showed no obvious toxicity on RAW264.7 cells. SSP-AK activated the production of NO, TNF-α, and IL-6 from RAW264.7 cells in a dose-dependent manner (Figure 5B–D). LPS did not induce a higher release of NO compared to the blank control, while SSP-AK samples with concentrations of 100–400 µg/mL significantly promoted the production of NO. At a concentration of 400 µg/mL, RAW264.7 cells produced 16.33 µM NO, which was 8-fold higher than that in the blank control and LPS (Figure 5B). In addition, the production of TNF-α in RAW264.7 cells on SSP-AK samples with concentrations of 100–400 µg/mL was 7824.41–8986.27 pg/mL, which was 17.2–19.8-fold higher than that in the blank control (Figure 5C). SSP-AK also promoted the production of IL-6 in RAW264.7 cells, ranging from 1900 to 4450 pg/mL (Figure 5D). NO, TNF-α, and IL-6 are considered pro-inflammatory factors that benefit human health, and the increased production of these pro-inflammatory factors in RAW264.7 cells indicated that SSP-AK exhibited satisfactory *in vitro* immunostimulatory activity, which could be explained by the high proportion of RG-I domain in the SSP-AK sample. A recent study confirmed that carrot RG-I fractions have remarkable immunomodulating activities that stimulate TNF-α, IL-6, IL-1β, IL-8, and IL-10 production in peripheral blood mononuclear cells, and the immunomodulating properties of RG-I are closely associated with the Mw and degree of esterification of RG-I [39]. Meanwhile, an *in vivo* study carried out in naive mice has also indicated that the immunomodulatory properties are related to the methyl esterification of the pectin HG domain [38]. The immunostimulatory activities of the SSP sample might be affected by the structural features of both the HG and RG-I domains, and specific evidence requires further investigation. Nevertheless, the data suggest that SSP-AK has the potential to be used as a healthy food ingredient.

## 4. Conclusions

In conclusion, our study successfully extracted a pectic polysaccharide (SSP-AK) from *Syzygium samarangense* fruit via alkaline treatment, achieving a 5.3% yield and 88.4% polysaccharide content. SSP-AK is predominantly an RG-I-rich pectin with a low degree of esterification (41.3%) and exhibits two molecular weight fractions (7658 kDa and 345.3 kDa). Notably, SSP-AK demonstrated excellent Ca^2+^-induced gelling properties. The results also indicated that SSP-AK gel formation was significantly affected by pectin concentration and Ca^2+^ concentration during Ca^2+^-induced gelation, including rheological behaviors, gel hardness, and water-holding capacity of SSP-AK gel. In addition, the SEM analysis revealed that the microstructure was significantly affected. Our findings indicate that SSP-AK exhibits remarkable *in vitro* antioxidant and immunomodulatory activities. These findings not only enhance our understanding of the structure-function relationships of *S. samarangense* polysaccharides but also highlight their potential as multifunctional ingredients in health-oriented food products.

## Figures and Tables

**Table 1:** Extraction yields, chemical components, degree of esterification, and monosaccharide compositions of *Syzygium samarangense* polysaccharide.

	Yield (%)	Polysaccharide (%)	Protein (%)	DE (%)	Monosaccharide Composition						Structure Content (mol%) ^a^			
					Rha	GluA	GalA	Glu	Gal	Ara	HG	RG-I	Rha/GalA	(Ara + Gal)/Rha
SSP-H	1.5 ± 0.1	82.1	1.5 ± 0.3	66.3 ± 0.3	1.0	0.6	4.7	2.7	8.9	5.2	15.9	69.4	0.2	14.1
SSP-AK	5.3 ± 0.2	88.4	5.5 ± 0.8	41.3 ± 0.3	1.0	0.4	4.3	1.3	4.1	3.5	22.6	64.8	0.2	7.6
SSP-AC	3.2 ± 0.3	80.5	3.2 ± 0.3	50.5 ± 0.7	1.0	0.6	6.5	2.5	8.9	5.4	21.9	65.6	0.2	14.3
SSP-DES	3.1 ± 0.1	76.3	3.1 ± 0.1	44.8 ± 0.5	1.0	0.3	1.3	2.1	3.9	3.7	2.6	77.6	0.8	7.5
SSP-AU	2.1 ± 0.1	83.2	2.1 ± 0.2	66.0 ± 0.6	1.0	0.4	9.7	1.2	5.4	3.6	40.6	51.5	0.1	9.0
SSP-U	0.8 ± 0.1	83.4	3.2 ± 0.4	51.1 ± 0.7	1.0	0.7	4.9	2.8	9.6	6.0	15.6	69.7	0.4	15.5
SSP-M	0.7 ± 0.1	81.3	4.1 ± 0.1	56.0 ± 0.7	1.0	0.7	2.4	2.0	9.9	5.6	6.3	80.3	0.2	15.6

^a^ The structural content is calculated in terms of molar percentages. HG and RG-I were homogalacturonan and rhamnogalacturonan-I, respectively. HG = GalA − Rha; RG-I = 2 Rha + Ara + Gal.

**Table 2:** Molecular size distribution of *Syzygium samarangense* polysaccharide.

	SSP-H	SSP-AK	SSP-AC	SSP-DES	SSP-AU	SSP-U	SSP-M
**Molecular weight, *M*_w_ (kDa)**							
Fraction 1	741.5 ± (0.8%)	7658 ± (0.9%)	1305 ± (0.7%)	533.1 ± (1.5%)	68.7 ± (0.9%)	605.3 ± (0.8%)	384.6 ± (1.0%)
Fraction 2	-	345.3 ± (2.8%)	44.2 ± (2.7%)	130.9 ± (1.5%)	-	-	-
Fraction 3	-	-	-	16.9 ± (6.4%)	-	-	-
**Mass fraction (%)**							
Fraction 1	100	15.7	8.4	6.3	100	100	100
Fraction 2	-	84.4	91.6	19.3	-	-	-
Fraction 3	-	-	-	74.4	-	-	-
***M*** **_w_** **/*M*_n_ (polydispersity)**							
Fraction 1	4.7	1.4	1.4	1.1	2.3	3.8	2.8
Fraction 2	-	2.0	1.9	1.2	-	-	-
Fraction 3	-	-	-	1.4	-	-	-

## Data Availability

The original contributions presented in this study are included in the article/Supplementary Materials. Further inquiries can be directed to the corresponding authors.

## Supplementary Materials

The following supporting information can be downloaded at: https://www.mdpi.com/article/10.3390/foods14071227/s1, Figure S1. (A–H) Size exclusion chromatograms and FT-IR spectra of SSPs prepared by different extraction techniques. Figure S2. (A) The flow behaviors at of SSPs prepared by different extraction techniques; (B) Time sweep of SSPs prepared by different extraction techniques; (C) Appearance of different Ca2+-induced SSP gels. (D) The flow behavior SSP gels.
