# Supplementary material for: Structural Characteristics, Gelling Properties, In Vitro Antioxidant Activity and Immunomodulatory Effects of Rhamnogalacturonan-I Rich Pectic Polysaccharides Alkaline-Extracted from Wax Apple (Syzygium samarangense)
Source: Foods. 2025 Mar 31;14(7):1227. doi: 10.3390/foods14071227 (PMC11988759; doi:10.3390/foods14071227)
Supplement: Supplementary file 1 [file foods-14-01227-s001.zip › foods-3536602-supplementary.pdf]

Supplementary contents

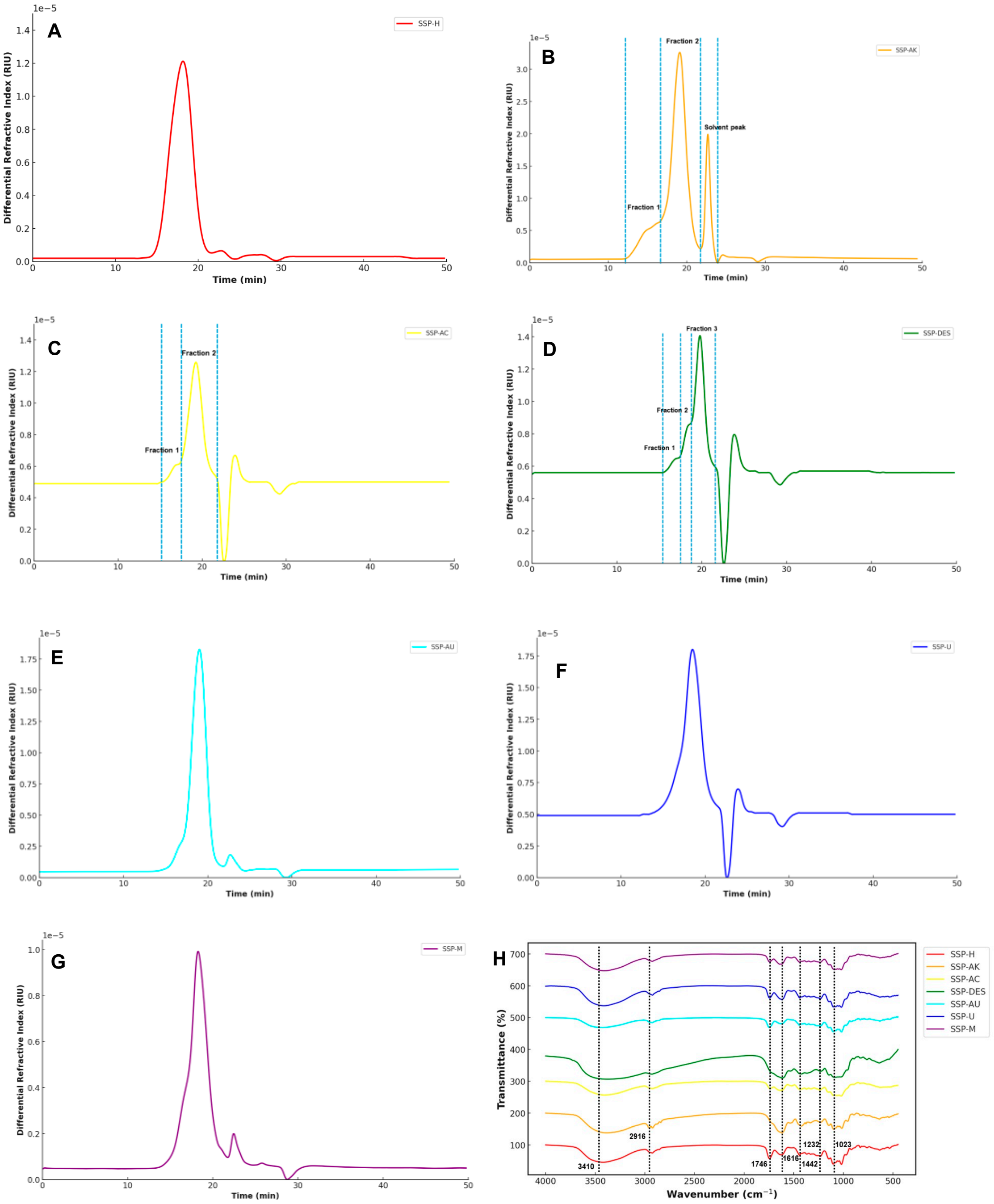

Figure S1. (A-H) Size exclusion chromatograms and FT-IR spectra of SSPs prepared by different extraction techniques.

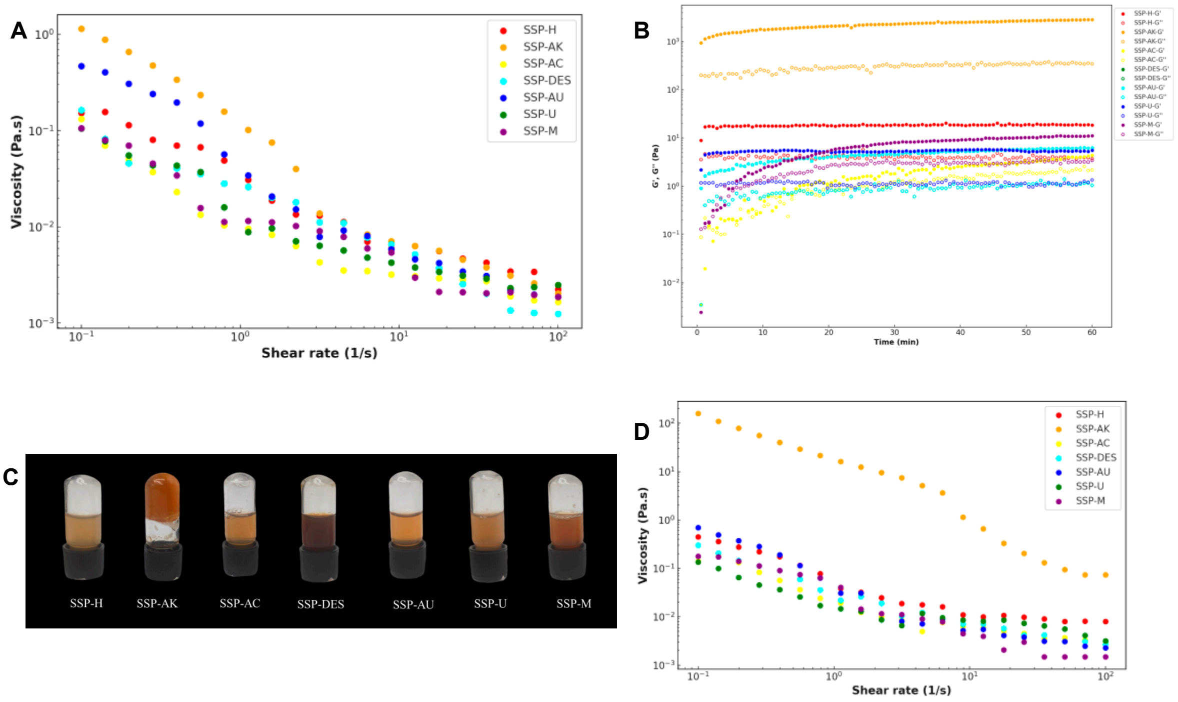

Figure S2. (A) The flow behaviors at of SSPs prepared by different extraction techniques; (B) Time sweep of SSPs prepared by different extraction techniques; (C) Appearance of different Ca^{2+}-induced SSP gels. (D) The flow behavior SSP gels.
